# Super-Toughed PLA Blown Film with Enhanced Gas Barrier Property Available for Packaging and Agricultural Applications

**DOI:** 10.3390/ma12101663

**Published:** 2019-05-22

**Authors:** Yuanping Jiang, Cong Yan, Kai Wang, Dawei Shi, Zhengying Liu, Mingbo Yang

**Affiliations:** College of Polymer Science and Engineering, State Key Laboratory of Polymer Materials Engineering, Sichuan University, Chengdu 610065, Sichuan, China; jypjiang@163.com (Y.J.); 18782027702@163.com (C.Y.); cngwbe8326@163.com (K.W.); missldyx@163.com (D.S.); liuzhying@scu.edu.cn (Z.L.)

**Keywords:** PLA, biodegradable films, toughness, film blowing

## Abstract

Polylactic acid (PLA) holds enormous potential as an alternative to the ubiquitous petroleum-based plastics to be used in packaging film and agricultural film. However, the poor viscoelastic behavior and its extremely low melt strength means it fails to meet the requirements in film blowing processing, which is the most efficient film processing method with the lowest costs. Also, the PLA’s brittleness and insufficient gas barrier properties also seriously limit PLA’s potential application as a common film material. Herein, special stereocomplex (SC) networks were introduced to improve the melt strength and film blowing stability of PLA; polyethylene glycol (PEG) was introduced to improve PLA’s toughness and gas barrier properties. Compared with neat poly(l-lactide) acid (PLLA), modified PLA is stable in the film blowing process and its film elongation at break increases more than 18 times and reaches over 250%, and its O_2_ permeability coefficient decreased by 61%. The resulting film material also has good light transmittance, which has great potential for green packaging applications, such as disposable packaging and agricultural films.

## 1. Introduction

Currently, the most widely used plastic products are films, especially packaging and agricultural films. Waste plastic films have brought about almost irreversible environmental damage and become a hot issue globally. Polylactic acid (PLA) has a lot of advantages, such as biodegradability, comprehensive performance, and compostability [1,2]. However, PLA can barely be used for industrial film blowing because of its low melt strength, brittleness, and low gas barrier property [3,4,5]. If the melt strength, toughness, and barrier property can be improved effectively, it is possible for PLA to be used in the film blowing process, therefore providing conditions for the industrial production and application of PLA film products.

Stereocomplex (SC) crystallites can be formed by blending poly(l-lactide) acid (PLLA) with poly(d-lactide) acid (PDLA), which has about a 50 °C higher melting point than neat PLA. The SC structure has been proved effective in improving the viscosity and crystallization ability of PLA [6,7]. PDLA-g-PEG-g-PDLA(DPD) triblock polymer even showed an easier formation of the SC and a faster crystallization rate of the PLLA matrix because the introduction of flexible PEG [8]. Despite much interesting research in regards to PLLA/PDLA and PLLA/DPD polymer blends [9,10,11], there are seldom reports about their film blowing capabilities or the film’s mechanical and barrier properties.

Polyethylene glycol (PEG) is a nontoxic additive with good biocompatibility. Normally, PEG can serve as a plasticizer and the direct blending of PEG with the PLA matrix can increase its toughness and crystallinity [12,13,14]. The flexible PEG chain can also help accelerate the crystallization of SC in PLLA/PDLA blends [15].

In this work, DPD and PEG are introduced to the PLLA matrix to improve PLA’s melt strength and toughness. By introducing DPD, we hope to form some SC crystallites in the melt blending process, which can improve PLA’s melt strength and film blowing stability. PEG can act as a plasticizer to help the crystallization of PLA and improve the toughness of the products. This study aims to obtain PLA blown film with good toughness and gas barrier property for packaging and agricultural applications.

## 2. Experiments and Characterizations

Materials. The applied PLLA (commercial, REVODE110, Zhejiang Haizheng Pharmaceutical Ltd., Taizhou, China, Mn = 5.0 × 10^4^ g/mol), DPD (lab-made as reported [8], the Mn of PDLA segment is 4135 g/mol, Mn of PEG segment is 4000 g/mol, Figure S1), PEG (Mn = 4000 g/mol, Aladdin Inc, Shanghai, China) were dried in a vacuum oven at 60 °C for 24 h before use. PE (thickness: 50 ± 5 µm) film was obtained from Chengdu Haihong Experimental Instrument Co. Ltd. (Chengdu, China).

Sample preparation. The PLLA, PLLA/DPD, and PLLA/DPD/PEG blends were prepared by melt blending at 175 °C for 5 min at 50 rpm using a Haake torque rheometer (XSS-300, Shanghai Kechuang Rubber Plastics Machinery Set Ltd., Shanghai, China). The mass ratio of PLLA: DPD was fixed at 9:1, then 5 wt% and 10 wt% PEG was added to PLLA/DPD system to prepare PLLA/DPD/PEG-5 and PLLA/DPD/PEG-10.

Extrusion blown PLA Films. Neat PLLA, PLLA/DPD, and PLLA/DPD/PEG-10 samples were prepared by melt compounding using a corotating twin-screw extruder (SHJ-20, Giant-China Mechanical and Electrical Co., Ltd, Nanjing, China). The process was carried out with a screw speed of 60 rpm and temperature profile between 170 °C and 195 °C. After that, an LSJ-10 single screw extruder with a screw diameter of 20 mm, L/D = 25, and an LF-250 film blowing machine (Labtech Engineering Co., Ltd, Samutprakarn, Thailand) was used to blow PLA films. Starting from the hopper to the die, the temperature profile of the extruder was set at 150–180–180–170 °C. The blow-up ratio was adjusted between 2.6–3.0, the screw speed was 26 Hz, and the draw rate was 2.8 m/min.

## 3. Characterization

Characterization of the melt blended samples. The thermal analysis of samples was performed with differential scanning calorimeter (DSC, Q20, TA Instruments Inc., New Castle, DE USA) under a nitrogen atmosphere from 20 °C to 250 °C at 10 °C/min. The crystallinity of PLA’s homocrystallites (X_cc_) and SC crystallites (X_sc_) can be calculated as follows:

(1) $$X_{\mathrm{cc}}={[(\Delta H}_{m}-{\Delta H}_{\mathrm{cc}})/{\Delta H}_{m}^{0}]\times 100\%;\mathrm{PLA}:{\Delta H}_{m}^{0}=93.6J/g$$

(2) $$X_{\mathrm{sc}}={\Delta H}_{\mathrm{sc}}/{\Delta H}_{m}^{0}\times 100\%;\mathrm{SC}-\mathrm{PLA}:{\Delta H}_{m}^{0}=142J/g$$

In the formulas, ${\Delta H}_{m}^{0}$ represents the melting enthalpy of 100% crystalline PLA, ΔH_m_ is the melting enthalpy, and ΔH_cc_ is the cold crystallization enthalpy of PLA homocrystallites measured via DSC. ΔH_sc_ is the SC crystallites’ melting enthalpy measured via DSC.

Each sample was also compression molded into 25 mm diameter and 1.5 mm thickness disks at 10 Mpa and 180 °C, and their dynamic rheological properties were tested on an AR2000ex rheometer (TA Instruments) with a parallel-plate geometry (diameter of 25 mm and 1100 µm in gap) at 170 °C under nitrogen atmosphere with angular frequency range from 0.0628 to 628 rad/s, and the applied strain was 0.1%.

Characterization of the blown films. The stress-strain measurements of the blown films were performed on a universal testing machine (5967, Instron, Norwood, MA, USA) using a 500 N load cell with a stretch speed of 5 mm/min under ambient conditions. The tensile fracture surfaces of blown films were coated with a thin layer of gold and observed by SEM (JEOL JSM-5900LV, JEOL PTE Ltd., Tokyo, Japan) at 5 kV. The optical absorption spectra of blown films (thickness: 50 ± 5 µm) were measured using a UV-3600 spectrophotometer (Shimadzu, Kyoto, Japan) over 300–800 nm. The oxygen permeability (P_O2_) of blown films were tested on a VAC-V2 film permeability testing machine (lab-think instruments, Jinan, China) at room temperature (23 ± 1 °C) with 50% relative humidity according to ISO2556:1974.

## 4. Results and Discussion

### 4.1. Thermal Behaviors of PLLA, PLLA/DPD, and PLLA/DPD/PEG Blends

**Figure 1:**
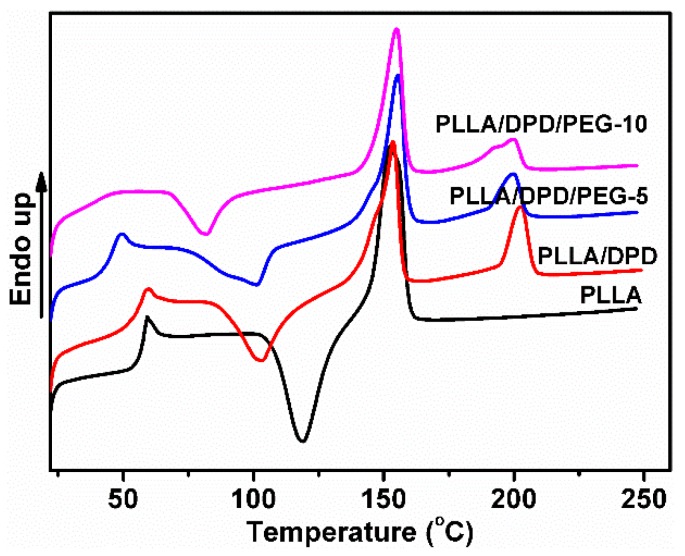
DSC curves of poly(l-lactide) acid (PLLA), PLLA/DPD, and PLLA/DPD/polyethylene glycol (PEG) blends.

As a melt enhancer, SC structure is very important in later stages of the PLA film blowing process. To study the SC crystallinity after common melt blending, the non-isothermal crystallization and melting behavior of neat PLLA, PLLA/DPD, and PLLA/DPD/PEG blends were studied by DSC, and the curves are shown in Figure 1. The PLLA homocrystallites’ cold crystallization temperature (T_cc_), cold crystallization enthalpy (ΔH_cc_), melting enthalpy (ΔH_m_), melting temperatures (T_m_) and crystallinity (X_cc_), and SC crystallites’ melting enthalpy (ΔH_sc_), melting temperatures (T_sc_), and crystallinity (X_sc_) are shown in Table 1. The T_m_ around 153 °C corresponding to α homocrystals can be observed in PLLA and PLLA/DPD. However, the higher T_m_ of homocrystals in PLLA/DPD/PEG blends at about 155 °C reflects an α polymorph of higher perfection [16]. Compared with neat PLLA, the T_m_ at about 200 °C appeared only in samples with DPD addition belongs to the SC structure, which was formed between the DPD’s PDLA segments and PLLA matrix. Compared with PLLA, PLLA/DPD showed a suppression effect to homocrystallization because of the introduction of SC crystallites. When PEG was added to PLLA/DPD, the PEG chains will accelerate the chain movement of PLLA chains and DPD chains. PLLA/DPD/PEG-5 sample showed an increase of X_cc_ and X_sc_ compared with PLLA/DPD, while PLLA/DPD/PEG-10 showed a slight decrease of X_sc_ because of more PEG contents.

### 4.2. Rheological Behavior of the PLLA Blends. 

The interfacial interaction between polymers can be evaluated by the variation of rheological parameters [17], such as storage modulus (G′), loss modulus (G″), complex viscosity, and relaxation time. The SC crystallites’ melt temperature is almost 50 °C higher than PLLA, which can serve as an efficient rheological modifier to improve the elastic response and viscosity of PLLA melt owing to the filler effect and crosslinking effect of the SC crystallite network [18,19].

**Figure 2:**
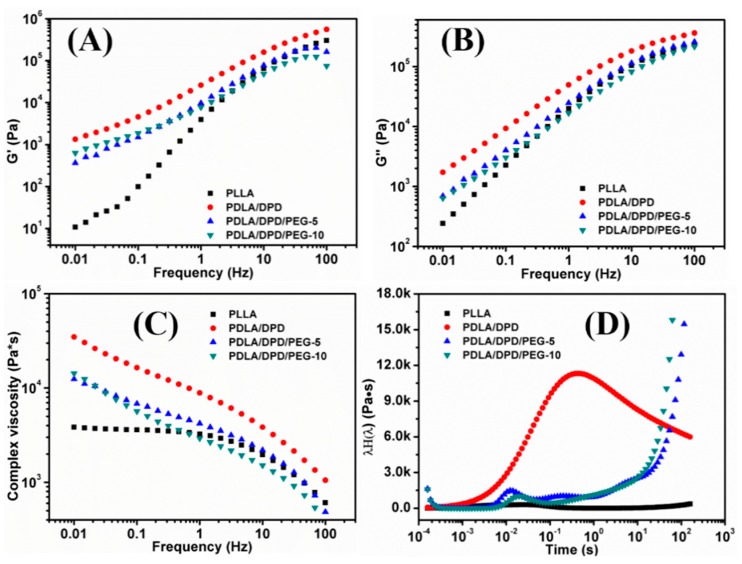
The G′ (**A**), G″ (**B**), complex viscosity (**C**) and continuous weighted relaxation (**D**) spectra of PLLA, PLLA/DPD, and PLLA/DPD/PEG measured by shear rheology.

The frequency sweep experiments of PLLA/DPD blends were carried out at 170 °C to investigate the effects of DPD and PEG on the rheological behaviors of the PLLA matrix. Figure 2 shows the G′ (Figure 2A), G″ (Figure 2B), complex viscosity (Figure 2C), and continuous weighted relaxation spectrum (Figure 2D) of samples.

**Figure 3:**
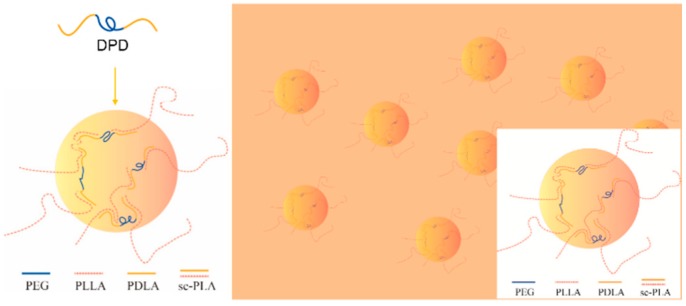
The mechanism of special SC networks formed between DPD and PLLA matrix.

The G′ and complex viscosity of PLA melt can reflect the change of melt strength to some degree. As shown in Figure 2, the G′, G″, and complex viscosity values increase significantly by adding DPD to the PLLA matrix, the G′ even has about two orders of magnitude increase at low frequency, which can help improve the melt stability in film blowing. The SC networks will remain unmelted at 170 °C and serve as fillers to improve the complex viscosity of the PLLA, which may be helpful to improve the melt strength in the film blowing of PLA. Adding plasticizer PEG to PLLA/DPD will directly reduce the melt strength compared with PLA/DPD, while with the help of DPD, the G′, G″, and complex values still showed an increase in low-frequency rate compared with neat PLLA. The PDLA segments in DPD chains can form SC structure with PLLA matrix, and the soft PEG chains in DPD may help the forming of SC structure and bind two SC crystallites together by chemical bonds to form more complicate SC networks in the system as assumed in Figure 3. The special SC network can be helpful to improve the melt strength in PLA’s film blowing processing.

The relaxation behavior is also closely related to the polymer’s melt strength and is very important in many processing methods, which determines the effect of processing parameters on material properties [20]. The relaxation time can reflect the chain entanglements at melt, which determines PLA’s melt strength. The continuous weighted relaxation spectrum (τH(τ)) of PLLA blends are calculated as below with G′ and G″ values obtained through frequency sweep testing. In the equation, τ is the relaxation time, ω is the angular frequency, and H(τ) is the relaxation time spectrum.

(3) $$G^{\prime }(\omega )=\int_{-\infty }^{+\infty }H(\tau )\frac{\omega^{2}\tau^{2}}{{1+\omega }^{2}\tau^{2}}\mathrm{dln}\tau$$

(4) $$G^{\prime \prime }(\omega )=\int_{-\infty }^{+\infty }H(\tau )\frac{\omega \tau }{{1+\omega }^{2}\tau^{2}}\mathrm{dln}\tau$$

As shown in Figure 2D, the PLLA’s longest relaxation time caused by the movement of free PLLA molecular segments or chains locates within the range of 0.02 s, which means PLLA chains lack entanglement and relax rapidly. After the addition of DPD, the intensity of the relaxation spectrum was enhanced and the longest relaxation time extends about 30 times longer to about 0.7 s. The special SC networks between DPD and PLLA chains and the interaction between the SC particles and matrix makes the movement of the PLLA chains more difficult, thus results in a longer relaxation time. The improvement of relaxation time can help increase the melt stability when the melt is extruded out of the die and blown to bubble in film blowing. Although the PLLA/DPD/PEG-5 and PLLA/DPD/PEG-10 showed a decrease in the intensity compared with PLLA/DPD, the characteristic relaxation peak of PLLA was still significantly improved and the relaxation peaks of SC crystallites appeared around 0.2 s and even longer. The longer relaxation time and higher relaxation intensity can also improve the melt stability and lead to a larger processing window of PLLA.

### 4.3. Film Blowing Process of PLLA, PLLA/DPD, and PLLA/DPD/PEG-10

Biodegradable PLA has great application potential in the field of packaging and agricultural films, which are mainly produced through film blowing. The stability of the bubble controlled by the melt strength of polymer is essential in the film blowing continuous production process. In this study, DPD products can effectively form special SC structure networks with PLLA chains in the matrix, which can enhance the melt viscosity and relaxation behavior of PLLA.

**Figure 4:**
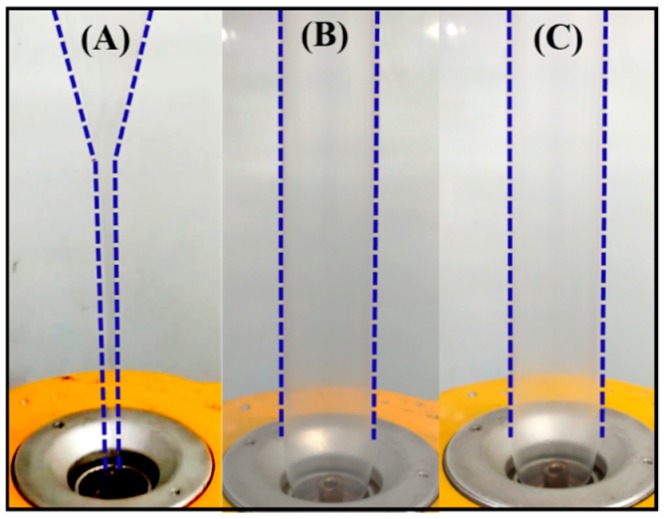
Film blowing bubbles of neat PLLA (**A**) and PLLA/DPD (**B**), and PLLA/DPD/PEG-10 (**C**).

The neat PLLA, PLLA/DPD, and PLLA/DPD/PEG-10 blends were chosen to conduct film blowing processing, and their blown bubble shapes were displayed in Figure 4 and Video S1. As shown in Figure 4A, PLLA’s low melt strength and quick relaxation character make it hard to meet the requirements of continuous film blowing process, and the unstable bubble burst, and bubble dancing appeared through the processing, which may disrupt the production and increase the production costs. With the appearance of melt enhancer special SC networks, the film blowing bubbles of PLLA/DPD (Figure 4B) and PLLA/DPD/PEG-10 (Figure 4C) are very stable, which can achieve continuous production of the film blowing process and lower the production costs of PLA film. The blow-up ratio can be maintained between 2.8–3.0, which is widely applied in industrial film blowing. The special SC structure (Figure S2) plays the main role in improving the melt stability of film blowing of PLLA, which helps achieve the continuous production of PLLA’s film blowing.

### 4.4. The Light Transmittance Properties of PLLA, PLLA/DPD, and PLLA/DPD/PEG-10 Films

**Figure 5:**
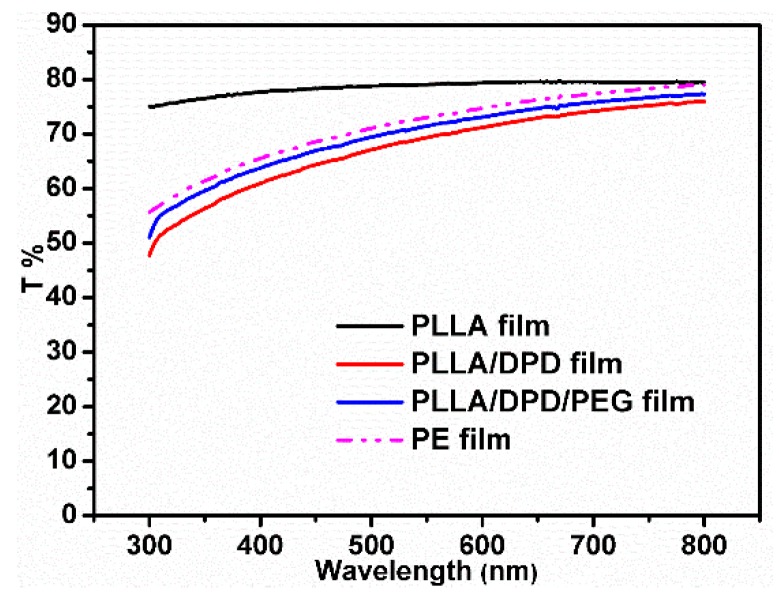
The light transmittance of blown films among 300–800 nm.

Light transmittance is very important in agricultural film production. It directly affects the growth of plants because the photosynthesis of plants mainly absorbs visible light at the wavelength of 400–700 nm [21,22]. Figure 5 and Table 2 show that the obtained PLLA/DPD and PLLLA/DPD/PEG-10 films hold less transparency compared with highly transparent PLLA films. However, PLLA/DPD/PEG-10 blown film is still highly transparent with a light transmittance property similar to PE, and its T% is 75.84% under 700 nm wavelengths of light. The PLLA/DPD/PEG-10 film can potentially be used as an agricultural film to ensure the solar transmittance through the film.

### 4.5. The Mechanical Properties of PLLA, PLLA/DPD, and PLLA/DPD/PEG-10 Films

**Figure 6:**
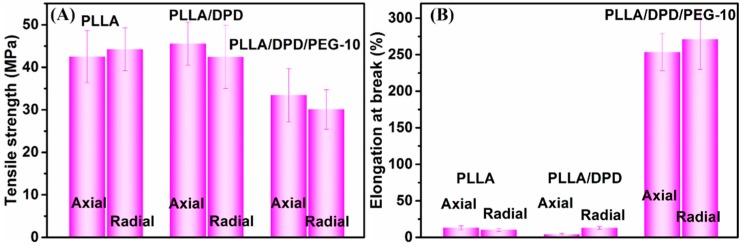
Tensile strength (**A**) and elongation at break (**B**) of blown films in the axial and radial direction.

**Figure 7:**
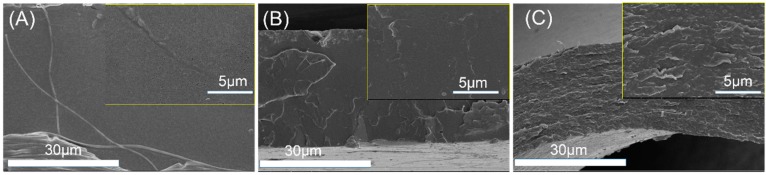
The morphology of tensile fracture surface of PLLA film (**A**) PLLA/DPD film (**B**) and PLLA/DPD/PEG film (**C**).

PLA’s application as film materials is also greatly limited by its brittleness [23,24]. In this work, PEG was added mainly to improve the toughness of PLLA film. After the film blowing process, the radial and axial direction of PLLA, PLLA/DPD, and PLLA/DPD/PEG-10 blown films were tested and their results were listed in Figure 6 and Table 3. The results show that PLLA film is a rigid material with a good tensile strength and poor toughness, which shows elongation at break value about 13.13% and 10.26% in the radial and axial direction. The PLLA/DPD film shows little change compared with the PLLA film, while the tensile strength of the PLLA/DPD/PEG-10 film decreased by about 21.39% in the radial direction, and 32.20% in the axial direction compared with PLLA film. The elongation at break of the PLLA/DPD/PEG-10 film reaches over 250% in both directions, increases by 18.3 times in the radial direction, and 25.4 times in the axial direction compared with the PLLA film. It also shows a much higher effectiveness compared with the other reported PLA/PBAT and PLA/PBS blown films at even higher loading [25]. As shown in Figure 7, the PLLA film and PLLA/DPD film exhibit smooth brittle fracture surfaces, which indicate their brittleness, while the PLLA/DPD/PEG-10 film’s rough surface demonstrates an obvious ductile fracture, indicating that the material has a good toughness. The super-toughed PLLA/DPD/PEG-10 film can potentially be used as packaging and agricultural films.

### 4.6. O_2_ Barrier Properties of Blown Films

**Figure 8:**
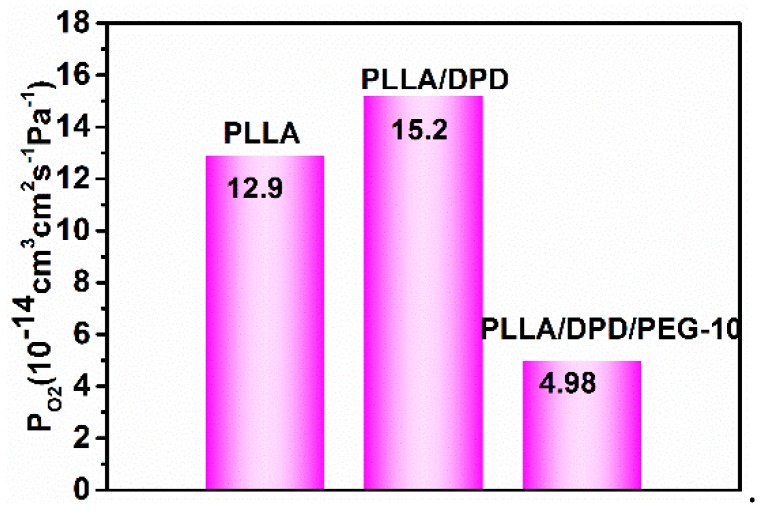
The permeability coefficient of O_2_ (P_O2_) of PLLA, PLLA/DPD, and PLLA/DPD/PEG-10 films.

The gas barrier property is very important as a packaging material, which holds the key for protecting vulnerable to O_2_ degradation of perishable goods [26,27]. As shown in Figure 8, the O_2_ permeability (P_O2_) is 12.9 × 10^−14^ cm^3^ cm^2^ s^−1^ Pa^−1^ in the PLLA film, which shows a poor gas barrier property compared with other films. With the addition of DPD, the PLLA/DPD is stable in the film blown process, but its O_2_ barrier ability was also impacted slightly, and its P_O2_ increased by about 23% to 15.2 × 10^−14^ cm^3^ cm^2^ s^−1^ Pa^−1^. With the addition of 10 wt% PEG to PLLA/DPD, the PLLA/DPD/PEG-10 can not only withstand the film blowing process but also achieve a 61% reduction in P_O2_ to 4.98 × 10^−14^ cm^3^ cm^2^ s^−1^ Pa^−1^. The O_2_ does not always diffuse along the direction perpendicular to the film and it may change its original permeability path from a vertical to horizontal direction [28]. In this case, an increase in oxygen permeability can be observed after the introduction of PEG to PLLA/DPD. The reasons can be inferred in two ways. Firstly, flexible PEG can fill up the interfacial defects between DPD and PLLA, and gives rise to the increasing path tortuosity and the decreasing cross section for O_2_ permeability. Secondly, more homocrystallites of higher perfection and the less permeable amorphous phase formed in the PLLA/DPD/PEG-10 film may also cause an increase of gas permeability [29]. In this work, the remarkable improvement in O_2_ barrier properties of PLLA/DPD/PEG-10 film makes it a good candidate for packaging material.

## 5. Conclusions

In this work, a comparative study was carried out in PLLA, PLLA/DPD, and PLLA/DPD/PEG blends to investigate their thermal and rheological properties. The special SC network proposed in this work was first used to improve the film blowing stability of PLLA. The addition of DPD can form melt enhancer SC crystallites within the PLLA matrix, which can form special SC networks to increase the melt strength of PLA, therefore, helping acquire stable blown bubble and conduct continuous film blowing process. To improve better mechanical and gas barrier performance, the addition of 10 wt% PEG to PLLA/DPD can greatly improve the toughness and gas barrier ability of PLA film without losing its film blowing stability. PLLA/DPD/PEG-10 film displays super toughness, good light transmittance, as well as better gas barrier property. The resulted biodegradable PLA film has a great potential in environmentally friendly packaging and agricultural applications.

## Figures and Tables

**Table 1:** Crystallization parameter of neat PLLA, PLLA/DPD, and PLLA/DPD/PEG blends.

Sample	ΔH_cc_ (J/g)	T_cc_ (°C)	ΔH_m_ (J/g)	T_m_ (°C)	ΔH_sc_ (J/g)	T_sc_ (°C)	X_cc_ (%)	X_c-sc_ (%)
PLLA	24.18	107.39	28.74	152.5	/	/	4.87	/
PLLA/DPD	18.33	103.34	20.71	153.5	7.78	203.01	2.54	5.48
PLLA/DPD/PEG-5	13.75	101.34	22.29	155.4	8.04	200.03	9.12	5.66
PLLA/DPD/PEG-10	13.27	82.19	19.62	154.9	6.64	200.11	6.78	4.67

**Table 2:** The light transmittance (T%) of films at different wavelengths of light.

Wavelength (nm)	300	400	500	600	700	800
PLLA	75.06	77.72	78.78	79.38	79.54	79.47
PLLA/DPD	47.77	60.95	67.1	71.19	74.21	75.95
PLA/DPD/PEG-10	51.04	63.75	69.42	73.1	75.84	77.28
PE	55.59	65.5	71.02	74.71	77.42	79.02

**Table 3:** Mechanical properties of neat PLLA, PLLA/DPD, and PLLA/DPD/PEG-10 blown films.

Sample	Tensile Strength (MPa)	Elongation at Break (%)
PLLA (Axial)	42.54 ± 6.13	13.13 ± 2.65
PLLA (Radial)	44.25 ± 5.05	10.26 ± 2.08
PLLA/DPD (Axial)	42.44 ± 7.41	4.34 ± 1.14
PLLA/DPD (Radial)	45.57 ± 5.05	12.89 ± 1.90
PLLA/DPD/PEG-10 (Axial)	33.44 ± 6.30	253.50 ± 25.41
PLLA/DPD/PEG-10 (Radial)	30.09 ± 4.65	270.91 ± 41.09

## Supplementary Materials

The following are available online at https://www.mdpi.com/1996-1944/12/10/1663/s1, Figure S1: 1H NMR spectrum of lab-made DPD (400 MHz, Chloroform-d), Figure S2: The XRD spectra of PLLA and PLLA/DPD/PEG-10 films, Video S1: Film blowing process of neat PLLA and PLLA/DPD/PEG.
