# Supplementary material for: Super-Toughed PLA Blown Film with Enhanced Gas Barrier Property Available for Packaging and Agricultural Applications
Source: Materials (Basel). 2019 May 22;12(10):1663. doi: 10.3390/ma12101663 (PMC6567083; doi:10.3390/ma12101663)
Supplement: Supplementary file 1 [file materials-12-01663-s001.zip › Supplementary Materials.pdf]

materials

Supplementary Materials: Super-Toughed PLA Blown Film with Enhanced Gas Barrier Property Available for Packaging and Agricultural Applications

Yuanping Jiang, Cong Yan, Kai Wang, Dawei Shi, Zhengying Liu and Mingbo Yang

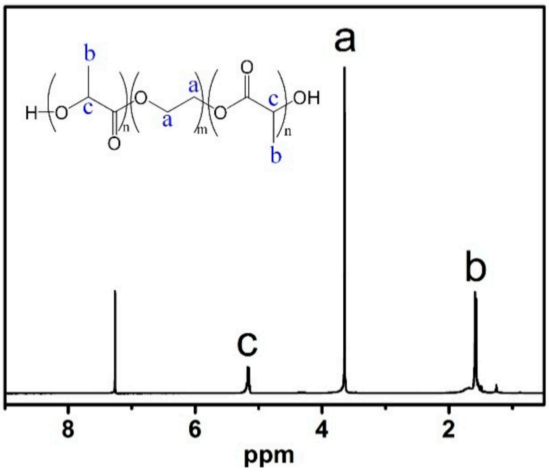

Figure S1. ^{1}H NMR spectrum of lab-made DPD (400 MHz, Chloroform-d).

The Mn of PEG segment applied was 4000 g/mol, and the PDLA segment was calculated to be 4135 g/mol with NMR results (AV III HD 400 MHz, Bruker Company, Leipzig, Switzerland).

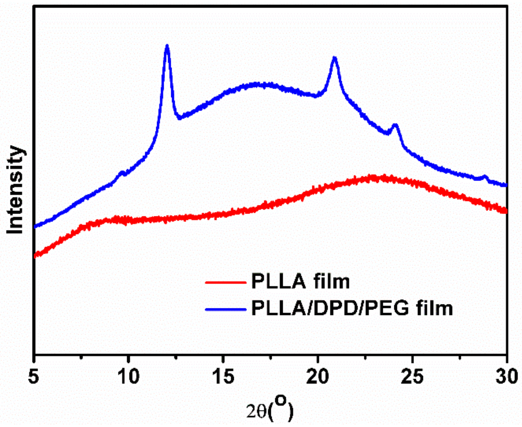

Figure S2. The XRD spectra of PLLA and PLLA/DPD/PEG-10 films.

XRD (ultima-IV, Rigaku, Tokyo, Japan) profiles was also added to illustrate SC in blown films. The PLLA film had no characteristic diffraction peaks because of its amorphous structure. However, the PLLA/DPD/PEG film showed obvious SC crystals' characteristic diffraction peaks (2θ= 12°, 21°, 24°).

© 2019 by the authors. Submitted for possible open access publication under the terms and conditions of the Creative Commons Attribution (CC BY) license (http://creativecommons.org/licenses/by/4.0/).
